# The complete chloroplast genome sequence of *Phlomoides kirghisorum* Adylov, Kamelin & Makhmedov 1987 (Lamiaceae), an endemic species of Fergana Valley

**DOI:** 10.1080/23802359.2023.2292159

**Published:** 2024-01-16

**Authors:** Min-Su Park, Nu-Ree Na, Chang-Gee Jang, Rustam Gulomov, Komiljon Tojibaev

**Affiliations:** a International Biological Material Research Center, Korea Research Instituto of Bioscience and Biotechnology, Daejeon, Republic of Korea; b Department of Biology Education, Kongju National University, Kongju, Republic of Korea; c Department of Biology, Namangan State University, Namangan, Republic of Uzbekistan; d Institute of Botany, Academy of Sciences of the Republic of Uzbekistan, Tashkent, Republic of Uzbekistan

**Keywords:** *Phlomoides kirghisorum*, complete chloroplast genome, phylogenetic analysis, Lamiaceae, Fergana valley

## Abstract

*Phlomoides kirghisorum* Adylov, Kamelin & Makhmedov 1987 is one of the poorly studied narrow endemics of Fergana Valley, one of Central Asia’s most densely human-populated regions. In this study, we sequenced, assembled, and characterized the complete plastome of *P. kirghisorum* by using high-throughput Illumina reads. The complete chloroplast genome consisted of 151,324 bp, including a large single-copy (LSC) region (82,775 bp), a small single-copy (SSC) region (17,357 bp), and two inverted repeat regions (25,596 bp each). In the chloroplast genome of *P. kirghisorum*, 133 genes were detected, comprising 88 protein-encoding genes, eight ribosomal RNA (rRNA) genes, and 37 transfer RNA (tRNA) genes. The phylogenetic analysis indicated that the genetic relationship between *P. kirghisorum* and *P. alpina* was very close. This study provides basic information to explore the molecular evolution of the *Phlomoides* genus and the Lamiaceae family.

## Introduction

The genus *Phlomoides* Moench (tribe Phlomideae, subfamily Lamioideae) is one of the largest genera of Lamiaceae, with approximately 150–170 species (Salmaki et al. 2012; Zhao et al. 2022). One of the major centers of species richness is located in Middle Asia (Lazkov 2011; Salmaki et al. 2012). The Conspectus Florae Asiae Media (Adylov and Makhmedov 1987) recorded 67 species. Later Lazkov (2016) and Sennikov and Lazkov (2013) described 9 more species. The last checklist of vascular plant species of Middle Asia (Khassanov 2015) shows the presence of at least 79 species of the genus. In Middle Asia, most species are geographically restricted to the Tian-Shan and Pamir-Alay mountains (Tojibaev et al. 2020).

**Figure 1.**
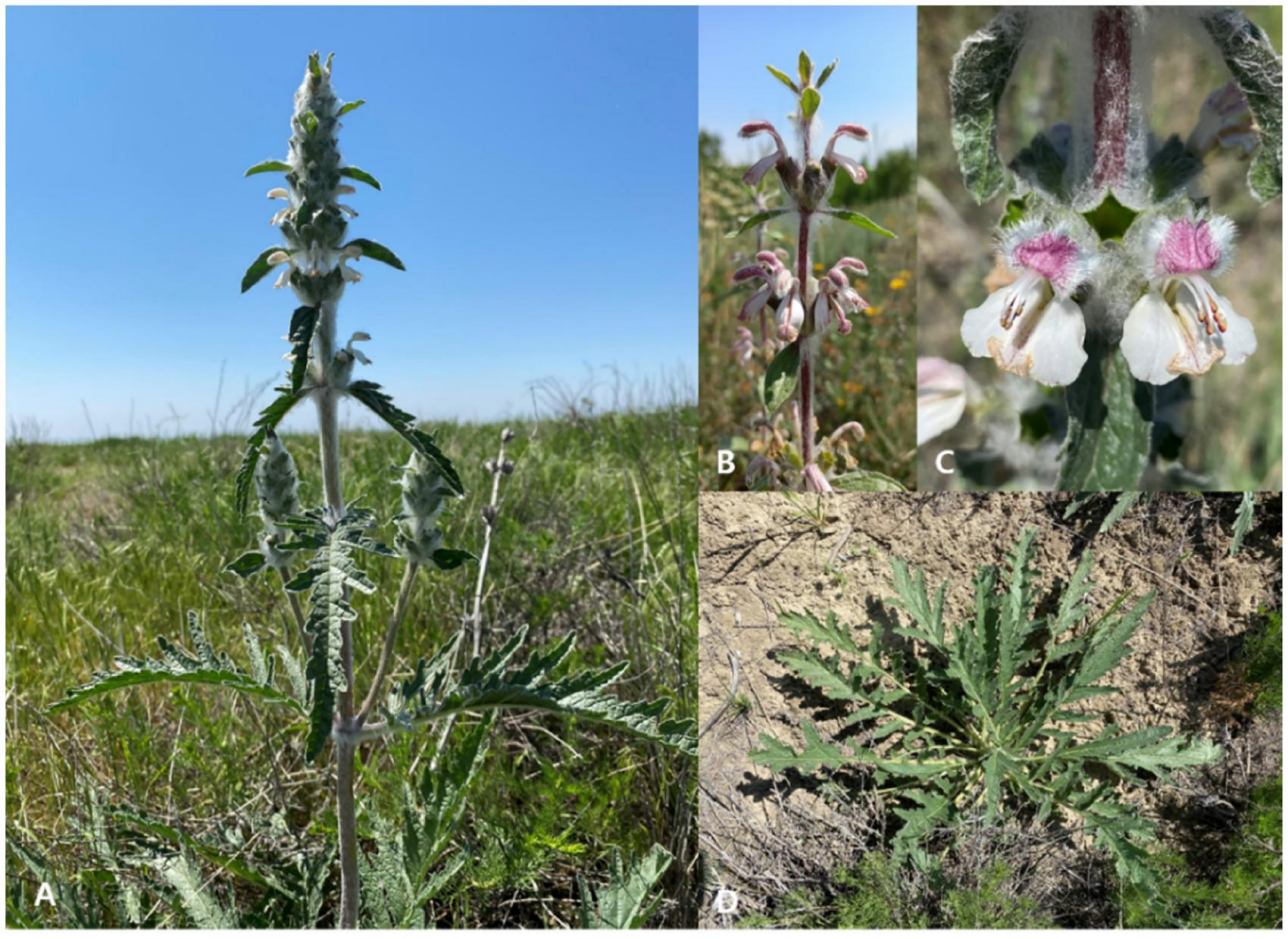
The morphological characteristics of *P. kirghisorum*. (A) Habit, (B) Inflorescence, (C) Flowers (oblong-ovate, oblong or broadly lanceolate corolla), (D) Basal leaves (pinnately parted). The photos taken by Rustam Gulomov.

*Phlomoides kirghisorum* Adylov, Kamelin & Makhmedov 1987 is one of the poorly studied narrow endemics of Fergana Valley (Uzbekistan and Kyrgyzstan), which is one of the most densely human-populated regions in Central Asia (Tojibaev et al. 2020). One of the major issues in the Fergana Valley is the conservation of natural landscapes with a diverse array of endemic and endangered species. Initially, the species (cited as *Eremostachys ferganensis* Ubuk.) was described based on specimens collected from the Bozbu-Too Mountains, Kyrgyzstan (Ubukeeva 1960). Adylov and Makhmedov (1987) reported this species in Kyrgyzstan, including the foothills and low mountains of Alay, Chatkal, and Fergana ridges. According to the latest data by Lazkov (2016), the species are known only from the type locality area. During field surveys in 2021, several plants belonging to this species were collected in Uzbekistan (Figure 1). The species can be easily distinguished from related species of *P. michaelis* by its corolla structure (oblong-ovate, oblong or broadly lanceolate) and leaf shape (pinnately parted and divided) (Lazkov 2016).

Sennikov and Lazkov (2013) presented the most updated subgeneric system of *Phlomides* and assigned these species to five sections which are more or less monophyletic. Several subsections and series are described in the genus, but their monophyly is highly under question by molecular phylogenetic data (Zohreh and Salmaki 2015). In this study, we report the complete chloroplast genome of *P. kirghisorum* and the phylogenetic tree in the *Phlomoides* genus. The chloroplast genome of *P. kirghisorum* provides basic genetic data that will help construct its phylogenetic relationships with related species.

## Materials and methods

The material was collected from Arbaghish village, Namangan province, Uzbekistan (41°16′39.6″N 71°54′21.9″E), and the voucher specimen was deposited at the Herbarium of the Kongju National University (KNH, voucher number: Gulomov R.K. #102, collected by Gulomov R.K., E-mail: gulomovr92@mail.ru), Kongju, Korea. Genomic DNA was extracted from dried leaf tissue using the DNeasy Plant Mini Kit (Qiagen, Seoul, South Korea). The DNA library was constructed with the TruSeq Nano DNA Kit (Illumina, San Diego, CA) and sequenced on the HiSeqXten platform (Macrogen, Seoul, South Korea). The sequencing generated 67,568,248 paired-end reads (2 × 151 bp) and low-quality sequences were filtered out by the FastQC tool (http://www.bioinformatics.babraham.ac.uk/projects/fastqc/). Then, the chloroplast genome was reconstructed using NOVOPlasty 4.1 (Dierckxsens et al. 2017), using a *P. bracteosa* partial *rbc*L gene sequence (ON947742.1) as seed. The chloroplast genome map, cis-splicing genes, and trans-splicing genes were generated using CPGview (Liu et al. 2023). The plastome was annotated in Geneious Prime 2023.1.1 (www.geneious.com), and the annotated plastome was submitted to GenBank (OR039725).

For phylogenetic analysis, 80 protein-coding regions were extracted from 14 chloroplast genomes of Lamioideae species with sequences of *Stachys sylvatica* L. as an outgroup. The extracted sequences were aligned using MAFFT v7.490 (Katoh and Standley 2013) embedded in Geneious Prime 2023.1.1. The jModelTest 2.1.10 was used to check the best model for the data matrix and the best model was GTR + I + G (Akaike information criteria) (Darriba et al. 2012). The maximum likelihood (ML) analysis was performed with RAxML v.8.0 (Stamatakis 2014) employing the GTR + I + G model, 1000 bootstrap replicates, and the rapid bootstrapping and search for the best-scoring ML tree algorithm.

## Results

**Figure 2.**
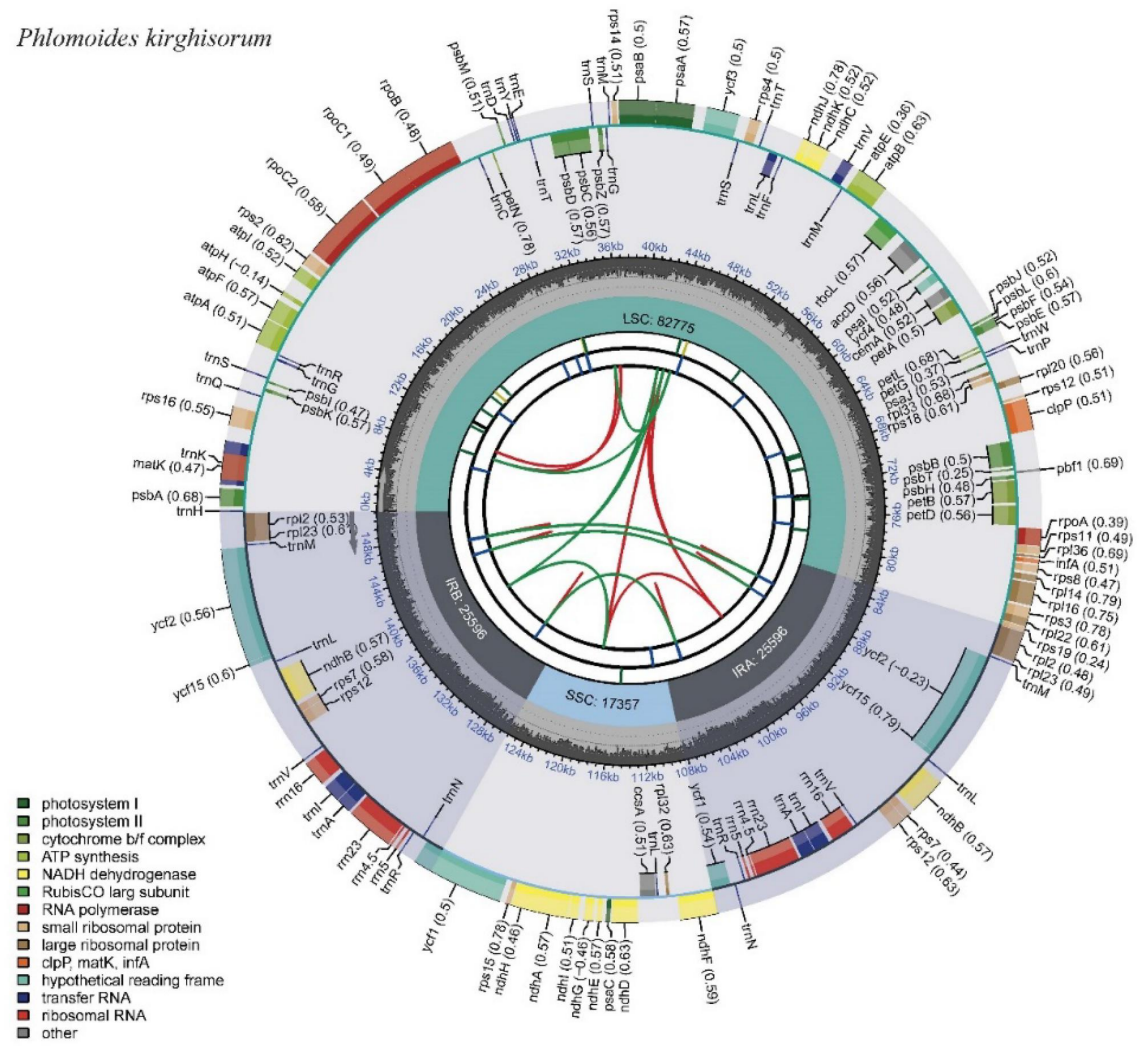
Chloroplast genome map of *P. kirghisorum*. The map contains six circles. From the center going outward, the first circle shows the distributed repeats connected with red (the forward direction) and green (the reverse direction) arcs. The next circle shows the long tandem repeats marked with short blue bars. The third circle shows the short tandem repeats (STRs) or microsatellite sequences as short bars with different colors. The fourth circle shows the size of the LSC, SSC, IRA, and IRB. The fifth circle shows the GC contents along the plastomes. The sixth circle shows the genes, and their optional codon usage bias is displayed in the parenthesis after the gene name. Genes are color-coded by their functional classification. The transcription directions for the inner and outer genes are clockwise and anticlockwise, respectively. The functional classification of the genes is shown in the bottom left corner.

**Figure 3.**
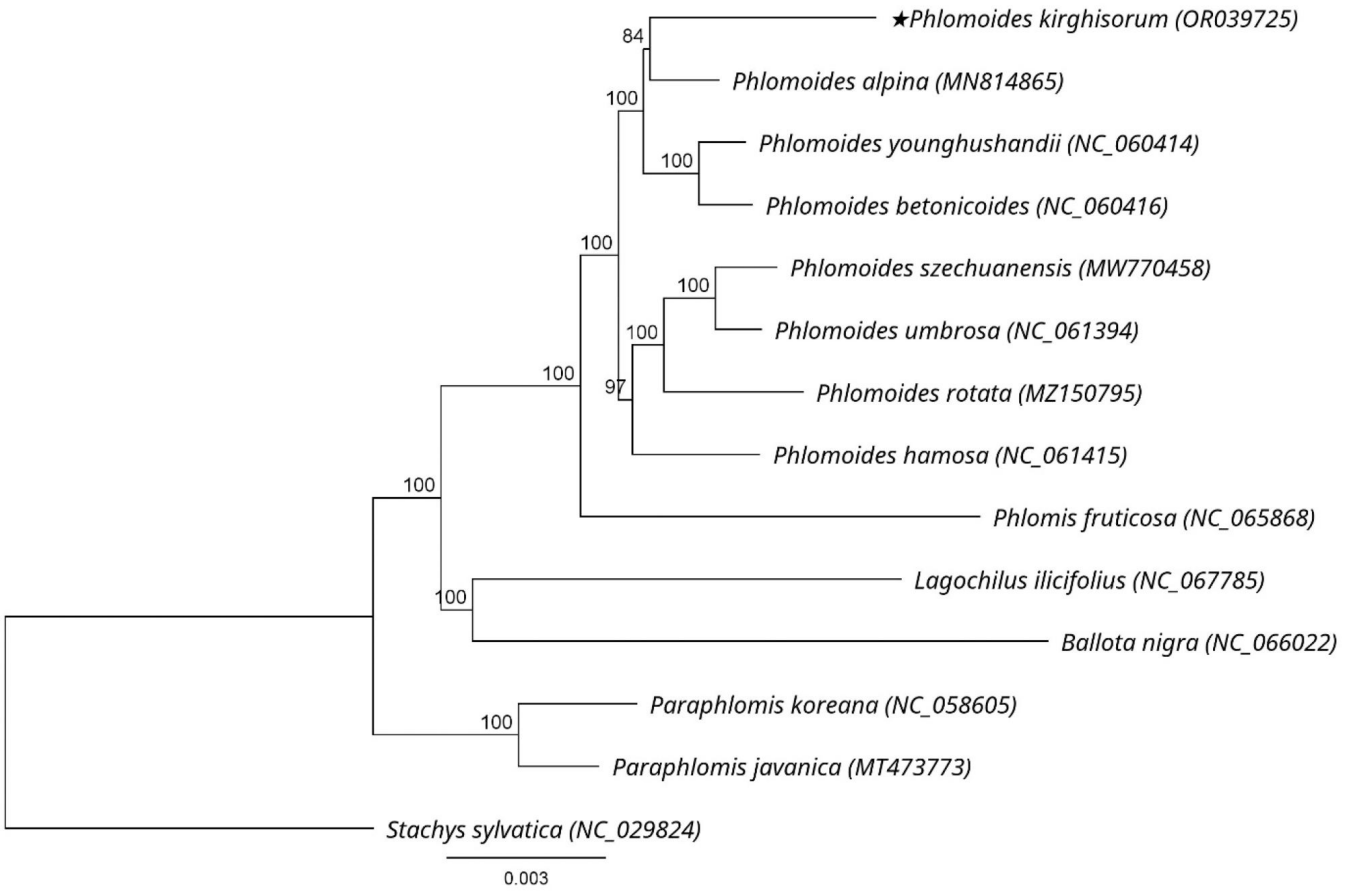
Phylogenetic tree of *P. kirghisorum* and 13 related species in Lamiaceae inferred from 80 protein-coding regions of chloroplast genomes using maximum likelihood methods. Note: the best substitution model was GTR + I + G (Akaike information criteria). Numbers in the nodes are bootstrap values with 1000 replicates. The scale bar means the expected number of nucleotide substitutions per site. The chloroplast genome of *Stachys sylvatica* was used as an outgroup. The following sequences were used: *P. alpina* MN814865 (Liu et al. 2020), *P. younghushandii* NC_060414 (Min et al. 2021), *P. betonicoides* NC_060416 (Zhao et al. 2019), *P. szechuanensis* MW770458 (unpublished), *P. umbrosa* NC_061394 (unpublished), *P. rotata* MZ150795 (Pema et al. 2021), *P. hamosa* NC_061415 (unpublished), *Phlomis fruticosa* NC_065868 (unpublished), *Lagochilus ilicifolius* NC_067785 (unpublished), *Ballota nigra* NC_066022 (unpublished), *Paraphlomis koreana* NC_058605 (unpublished), *Paraphlomis javanica* MT473773 (Zhao et al. 2021), *Stachys sylvatica* NC_029824 (unpublished).

A total of 4,128,387 reads were mapped in Geneious to check the assembled plastome, yielding a coverage of 4105× (Supplementary Figure 1). The chloroplast genome of *P. kirghisorum* showed a typical quadripartite structure consisting of two reverse repeated regions (IRa and IRb) of 25,596 bp in length, separated by a large single-copy region (LSC: 82,775 bp) and a small single-copy region (SSC: 17,357 bp). It had an overall 38.5% GC content and encoded 133 genes including 88 protein-coding genes (PCGs), eight ribosomal RNA genes (rRNAs), and 37 transfer RNA genes (tRNAs) (Figure 2). Thirteen genes including *rps*16, *atp*F, *rpo*C1, *ycf*3, *clp*P, *pet*B, *pet*D, *rpl*16, *rpl*2, *ndh*B, *ndh*A, *ndh*B, and *rpl*2 are cis-splicing genes (Supplementary Figure 2(A)), and the *rps*12 is a trans-splicing gene (Supplementary Figure 2(B)). The genus *Phlomoides* can be divided into two groups based on the results of phylogenetic research using maximum likelihood analysis. The phylogenetic position of *P. kirghisorum* was found to be the sister of *P. alpina* (Figure 3).

## Discussion and conclusions

The complete chloroplast genome of *P. kirghisorum* was first sequenced and found to exhibit a total length of 151,324 bp. There were no significant differences in genome size or gene content of *P. kirghisorum* compared to other chloroplast genomes in *Phlomoides* (Zhao et al. 2019; Liu et al. 2020; Min et al. 2021; Pema et al. 2021; Zhao et al. 2021). The close relationship between *P. kirghisorum* and *P. alpina* can provide a reference for studying the phylogenetic relationship of the whole genus. However, the analysis was conducted using only 9 complete cpDNAs of Phlomideae members and 80 protein-coding regions. Therefore, more research is needed to gain a deeper understanding of the evolution of this tribe, taking into account more samples, morphology, biogeography, and other evidence. In conclusion, the recently completed cpDNA of *P. kirghisorum* will provide meaningful information for developing molecular markers, tracing evolutionary history, and reconstructing the phylogeny of *Phlomoides*, Phlomideae tribe, and the Lamiaceae family.

## Supplementary Material

Supplemental Material

## Data Availability

The genome sequence data that support the findings of this study are openly available in GenBank of NCBI at (https://www.ncbi.nlm.nih.gov/) under the accession no. OR039725. The associated BioProject, SRA, and Bio-Sample numbers are PRJNA807820, SRR18131954, and SAMN26001778 respectively.
