# Supplementary material for: The complete chloroplast genome sequence of Phlomoides kirghisorum Adylov, Kamelin & Makhmedov 1987 (Lamiaceae), an endemic species of Fergana Valley
Source: Mitochondrial DNA B Resour. 2024 Jan 16;9(1):104–8. doi: 10.1080/23802359.2023.2292159 (PMC10795634; doi:10.1080/23802359.2023.2292159)
Supplement: Supplemental Material [file TMDN_A_2292159_SM5297.docx]

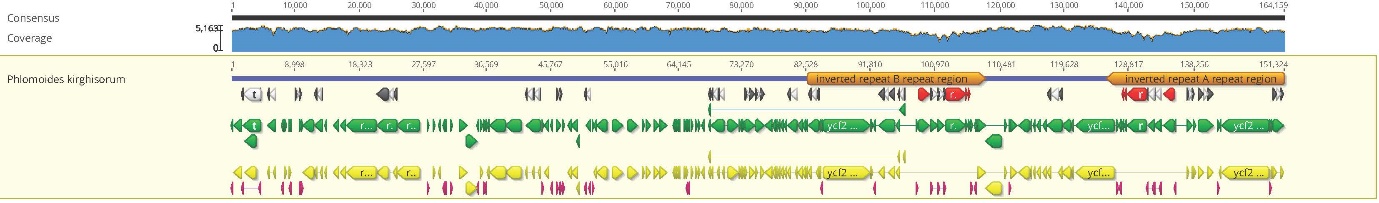


Supplementary Figure S1. Clean reads coverage depth map of *Phlomoides kirghisorum*.


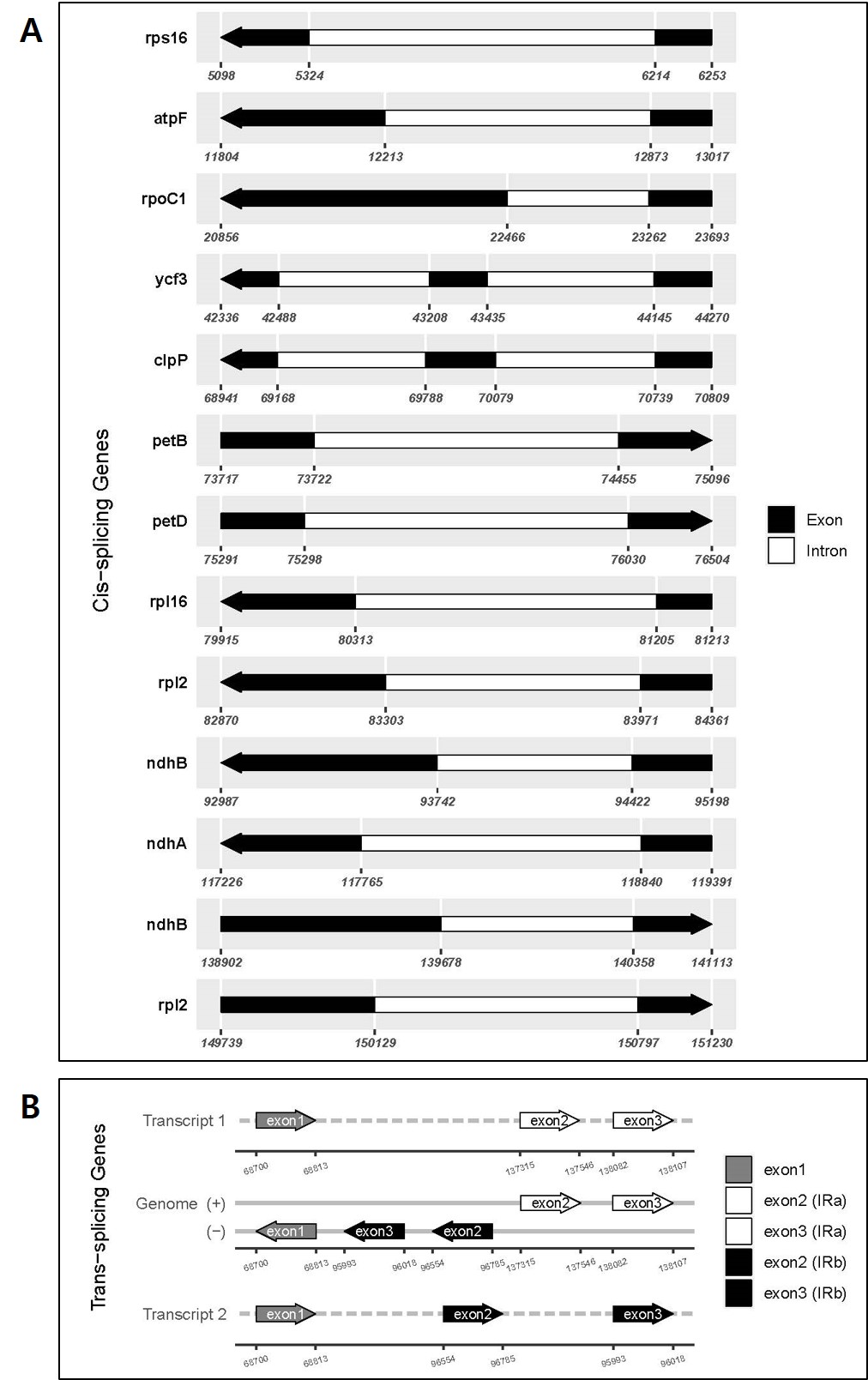


Supplementary Figure S2. Schematic of the cis-splicing gene (A) and trans-splicing genes (B) maps generated for the chloroplast genome of *Phlomoides kirghisorum*.
